# Effects of Prenatal Hypoxia on Nervous System Development and Related Diseases

**DOI:** 10.3389/fnins.2021.755554

**Published:** 2021-10-25

**Authors:** Bin Wang, Hongtao Zeng, Jingliu Liu, Miao Sun

**Affiliations:** Institute for Fetology, The First Affiliated Hospital of Soochow University, Suzhou, China

**Keywords:** prenatal hypoxia, nervous system, development, behavior, mechanism

## Abstract

The fetal origins of adult disease (FOAD) hypothesis, which was proposed by David Barker in the United Kingdom in the late 1980s, posited that adult chronic diseases originated from various adverse stimuli in early fetal development. FOAD is associated with a wide range of adult chronic diseases, including cardiovascular disease, cancer, type 2 diabetes and neurological disorders such as schizophrenia, depression, anxiety, and autism. Intrauterine hypoxia/prenatal hypoxia is one of the most common complications of obstetrics and could lead to alterations in brain structure and function; therefore, it is strongly associated with neurological disorders such as cognitive impairment and anxiety. However, how fetal hypoxia results in neurological disorders remains unclear. According to the existing literature, we have summarized the causes of prenatal hypoxia, the effects of prenatal hypoxia on brain development and behavioral phenotypes, and the possible molecular mechanisms.

## Introduction

**FIGURE 1:**
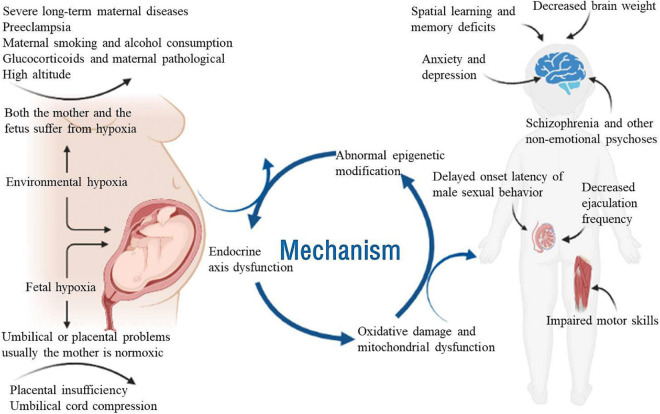
Schematic diagram of the effects of prenatal hypoxia on neurological development and related diseases. Adversity during pregnancy, such as chronic maternal illness, preeclampsia, high-altitude pregnancy, placental dysfunction, and umbilical cord compression, can cause prenatal hypoxia. Adequate oxygen is essential for the growth and development of the fetus. Prenatal hypoxia affects fetal growth and development in terms of epigenetics, endocrine axis dysfunction and mitochondrial oxidative damage. Prenatal hypoxia also causes dysfunction of the offspring after birth, such as learning and memory disorders, male sexual dysfunction, motor dysfunction and emotional disorders.

The whole process of pregnancy and delivery is critical for the healthy development of the fetus. Therefore, limited oxygen supply (hypoxia) during pregnancy remains one of the greatest threats to fetal development (Piesova and Mach, 2020). The overall incidence of fetal hypoxia varies greatly among European hospitals, ranging from 0.06 to 2.8% (Giannopoulou et al., 2018). Although fetal cells and organs have some compensatory responses to hypoxia, they are insufficient to protect the developing brain from severe or chronic hypoxia (Riljak et al., 2016; Piesova et al., 2020). Prenatal hypoxia decreases the number of neurons and synaptic density in the hippocampus, which also alters the release of neurotransmitters (Chen et al., 2020; Camm et al., 2021). Prenatal hypoxia can have adverse effects on the development of the central nervous system (CNS) and may lead to future behavioral disorders. In various studies, offspring exposed to prenatal hypoxia showed dysfunctional changes in brain development, movement, memory and emotion (Giannopoulou et al., 2018; Duran-Carabali et al., 2019; Zhuravin et al., 2019; Piesova and Mach, 2020). Therefore, it is crucial to study the effects of prenatal hypoxia using animal models that can replicate human embryonic development, especially fetal brain development. In this review, we focus on the causes of prenatal hypoxia and the effect of prenatal hypoxia on brain development, synaptic plasticity and behavior and summarize the possible mechanisms involved (Figure 1).

## Prenatal Hypoxia

### Cause of Prenatal Hypoxia

Prenatal hypoxia caused by abnormal pregnancy, maternal infection and labor can affect the developmental and behavioral characteristics of animals (Gabrielli et al., 2012). According to the cause of prenatal hypoxia, hypoxia can be divided into two types: environmental hypoxia due to changes in the external or maternal environment, in which both the mother and the fetus suffer from hypoxia, and fetal hypoxia. It may be caused by umbilical or placental problems, and usually the mother is normoxic (Piesova and Mach, 2020).

Factors leading to intrauterine hypoxia include severe long-term maternal diseases (including heart, lung and kidney diseases), pregnancy with anemia, hemoglobinopathy, placental insufficiency, maternal infection, umbilical cord compression, alcohol consumption, maternal smoking, preeclampsia, and administration of glucocorticoids (Sandau and Handa, 2007; Gabrielli et al., 2012; Gonzalez-Rodriguez et al., 2014; Gumusoglu et al., 2020; Piesova and Mach, 2020). Altitudes above 2,500 m are considered high altitude, which is also one of the reasons for prenatal hypoxia. There are approximately 140 million people living in high-altitude environments worldwide, compared with sea level. Pregnancy at high altitude may lead to a significant decrease in maternal arterial pO_2_ and affect placental growth (Zamudio, 2003; Patterson and Zhang, 2010).

When there is a brief hypoxia attack to the mother during pregnancy, the uterine artery contracts or compresses, which will reduce the amount of oxygen that is delivered to the placenta. The placenta is the main interface between the mother and the fetus, and its development and placental oxygen consumption also affect fetal oxygen supply. In addition, changes in maternal pO_2_ and/or abnormal placental development or metabolism may reduce pO_2_ in the fetal arteries and lead to fetal hypoxia (Jensen et al., 1999; Myatt, 2006).

### Animal Models Used for Prenatal Hypoxia Studies

In recent years, to better understand the effects of prenatal hypoxia on brain development and the molecular mechanism in offspring, animal models of prenatal hypoxia have been widely used and reviewed, including rats, mice, rabbits, chickens, and sheep (Table 1).

**TABLE 1:** Animal models used for prenatal hypoxia studies.

**Species**	**Method**	**Offspring influence**	**References**
Human	Autopsy of the human neonate to perinatal hypoxia/ischemia	Neurological and/or cognitive deficits, dopaminergic neurotransmitter dysfunction	Giannopoulou et al., 2018
Human	Not mentioned	Cognitive disorders, Alzheimer’s disease	Nalivaeva et al., 2018
Human	Not mentioned	Attention-deficit/hyperactivity disorder	Getahun et al., 2013; Owens and Hinshaw, 2013
Rat	Exposure to acute hypoxia on day 14 of pregnancy	The number of synaptopodin-positive dendritic spines was reduced, learning and memory deficits	Vasilev et al., 2016; Zhuravin et al., 2019
Rat	Uterine artery ligation on the 16th day of pregnancy	Pax6 immunoreactivity showed diverse patterns in the neurogenic zone	So et al., 2017
Rat	Pregnant female rats were injected with 5, 25, and 50 mg/kg sodium nitrite	Impaired spatial memory	Sosedova et al., 2019
Rat	Unilateral ligation of uterine artery at E17	White and gray matter damage, myelin loss, motor, sensorimotor and short-term memory deficits	Delcour et al., 2012b
Rat	Clamping of the uterine vascular system of pregnant rats at 17 days gestation for 30 min	Learning deficit	Cai et al., 1999
Rat	(E14 or E18) subjected to acute normoxia (7% O_2_ within 3 h)	Physiological development and formation of motor behavior delayed, learning ability and memory impaired	Dubrovskaia and Zhuravin, 2008
Rat	Pregnant rats treated with hypoxia (10.5% O_2_) on days 4–21 of pregnancy	Impaired learning and memory ability, spatial acquisition, and retrieval deficits	Wei et al., 2016
Rat	Transient occlusion of uterine perfusion	Hippocampal injury and cognitive defects, induced both mitochondrial impairment and mitochondrial biogenesis	Jia et al., 2020
Rat	4 h/day throughout pregnancy (E1–E21)	Sex-dependent anxiety-like behavior	Wang et al., 2013
Rat	On the 18th day of pregnancy, the maternal uterine artery was blocked with aneurysm clamp for 45 min	Increased anxiety-like behavior and lack of habits and a lack of habituation	Sab et al., 2013
Mouse	Mice were exposed to systemic hypoxia at E19.5 (O_2_ fraction of 6% for 6 h)	Neuronal apoptosis.	Chen et al., 2015
Mouse	Mice were placed in a Plexiglas chamber filled with a mixture of 9% O_2_, 3% CO_2_, and balanced N_2_ for 2 h	High susceptibility of neonatal seizures and epilepsy, impaired migration signaling, motor disabilities, and memory damage in adult offspring	Golan et al., 2004, 2009; Louzoun-Kaplan et al., 2008
Sheep	The fetus was intubated through the umbilical cord vasculature to a pumpless extracorporeal oxygenator for 22 ± 2 days in mid-gestation.	Increase of white matter vessels, the decrease of neuronal density and the damage of myelination	Lawrence et al., 2019
Sheep	Fetal lambs (111 ± 3 days) were oxygen delivery was limited by the umbilical circuit oxygenator maintained in the artificial womb for a mean of 22 ± 6 days	Altered cerebrovascular resistances and loss of brain mass	McGovern et al., 2020
Sheep	Pregnant ewes undergo a sterile procedure between 88 and 92 days of gestational age to control blood flow to the brain through the common carotid artery and vertebral artery.	Destroy the dendration and activity of neurons in the subplate of preterm fetal sheep	McClendon et al., 2017
Sheep	Pregnant sheep received instrumental treatment at 101 ± 1 days, and preterm instrumental fetal sheep received 25 min of umbilical cord occlusion at 0.7 pregnancy	Profound brain inflammation and mobilization of the peripheral innate immune system	Jellema et al., 2013
Sheep	Pregnant sheep were surgically instrumented at gestational day 122 ± 3 days, the umbilical cord was compressed until the fetal arterial partial pressure of oxygen dropped to 50% for 30 min, and the hypoxia treatment was repeated every day for 5 days	Reduced the vascular agonist-mediated contraction in fetal middle cerebral arteries	Su et al., 2020
Chick	During the 21 day incubation, 50% of the eggshell was covered with impermeable membrane for 4 or 8 days from day 14 and day 10 to limit the gas exchange of the eggshell	Memory impairment	Camm et al., 2001
Chick	On days 10–18 or 14–18 of incubation, half-wrap the eggs in impermeable membrane	Cognitive impairment	Camm et al., 2005
Rabbit	Uterine ischemia lasting 30, 37, 38, or 40 min at 21–22 days gestation	Hypertonia and abnormalities in motor control	Derrick et al., 2004
Guinea pig	Unilateral uterine artery ligation on 30–32 days of pregnancy	Decreased survival of neurons in the cerebral cortex	Chung et al., 2015
Guinea pig	On GD 52, pregnant females were placed in a plexiglass chamber containing 10.5% O_2_ for 10 days	Decreased the density of NeuN-immunoreactive neurons in the CA1 region of the hippocampus	Blutstein et al., 2013

For humans, a growing number of clinical, epidemiological, and experimental studies in recent years have demonstrated that maternal hypoxia plays a critical role in brain development and postnatal life, which increases vulnerability to later development of neuropsychiatric and neurodegenerative diseases, including ADHD, depression, anxiety, Parkinson’s disease, and Alzheimer’s disease (Nalivaeva et al., 2018). Around the world, 11.1% of live births worldwide are premature (born before 37 weeks of gestation) each year, and this proportion appears to be increasing (Blencowe et al., 2013). Prenatal hypoxia seriously affects the growth and development of the fetus, leading to low birth weight, premature birth and ischemic hypoxic encephalopathy, and severe cases can directly lead to fetal asphyxia death (Bennet et al., 2012). The incidence of neonatal brain injury in preterm infants is high, and has adverse effects on motor, social, behavioral, cognitive, attention, and sensory outcomes (Getahun et al., 2013; Owens and Hinshaw, 2013; Gopagondanahalli et al., 2016; Giannopoulou et al., 2018; Nalivaeva et al., 2018).

Rats and mice are the most popular and convenient rodent models, and both can be used in prenatal hypoxia studies. In rats, when hypoxia was induced by unilateral uterine artery ligation on the 16th day of pregnancy, Pax6, which plays an important role in neurogenesis, showed diverse patterns in the neurogenic zone (So et al., 2017). In addition, different rat models of prenatal hypoxia showed different degrees of learning and memory deficits (Cai et al., 1999; Dubrovskaia and Zhuravin, 2008; Delcour et al., 2012b; Vasilev et al., 2016; Wei et al., 2016; Sosedova et al., 2019; Zhuravin et al., 2019; Jia et al., 2020). Prenatal hypoxia (4 h/day throughout pregnancy) induces sex-dependent demethylation of the CpG site of the Crhr1 gene in the male promoter region, resulting in increased Crhr1 mRNA in the PVN region and anxiety-like behavior in adult offspring (Wang et al., 2013). On the 18th day of pregnancy, the maternal uterine artery was blocked with an aneurysm clip for 45 min, and the resulting prenatal hypoxia was also characterized by increased anxiety behavior and lack of habituation (Sab et al., 2013). In mice, gestation of wild-type C57BL/6J mice exposed to systemic hypoxia (O_2_ fraction of 6% for 6 h) at E19.5 increased the expression of c-myc, leading to increased Apaf-1 levels through inhibition of miR-23b-27b cluster expression, which enhanced the sensitivity of neurons to apoptosis (Chen et al., 2015). Pregnant mice were placed in a Plexiglas chamber filled with a gas mixture of 9% oxygen, 3% carbon dioxide and balanced nitrogen for 2 h, which induced a decrease in key protein levels of the GABA pathway in the cerebral cortex and may lead to high susceptibility to neonatal seizures and epilepsy, impaired migration signaling in the hippocampus, motor disabilities and memory damage in adult offspring (Golan et al., 2004, 2009; Louzoun-Kaplan et al., 2008).

Sheep, as one of the large experimental animals, is considered the most suitable model for fetal studies because the fetal reaction can be observed directly through recording electrocorticograms (ECOGs) *in utero* (Lacan et al., 2020). Mid-gestation fetuses were intubated through the umbilical cord vessels in a pumpless extracorporeal oxygenator and supported for 22 ± 2 days under hypoxic conditions in a fluid-filled environment, leading to an increase in white matter vessels, a decrease in neuronal density and damage to myelination (Lawrence et al., 2019). Fetal lambs (111 ± 3 days) with oxygen delivery were limited by the umbilical circuit oxygenator maintained in the artificial womb for a mean of 22 ± 6 days, leading to altered cerebrovascular resistances and loss of brain mass (McGovern et al., 2020). Pregnant ewes bred in time underwent aseptic surgery between 88 and 92 days gestational age to control the flow of blood to the brain through the common carotid and vertebral arteries and to disrupt maturation of arborization and activity in preterm fetal ovine subplate neurons (McClendon et al., 2017). Preterm instrumented fetal sheep were exposed to 25 min of umbilical cord blockade at 0.7 gestation, resulting in profound microglial activation and proliferation in the hippocampus, periventricular and subcortical white matter, leading to severe brain atrophy, hypomyelination, region-specific preOL vulnerability, and continuously inhibited brain function, providing evidence of global hypoxia-ischemia leading to profound brain inflammation and mobilization of the peripheral innate immune system (Jellema et al., 2013). Pregnant sheep were surgically instrumented at gestational day 122 ± 3 days, the catheters were inserted into the fetal femoral arteries on both sides, and an inflatable occluder was placed around the umbilical cord. Five to seven days after the operation, the umbilical cord was compressed to reduce the umbilical blood flow until the fetal arterial partial pressure of oxygen dropped to 50% of the baseline lasted for 30 min, and the hypoxia treatment was repeated every day for 5 days. It was found that prenatal hypoxia reduced the vascular agonist-mediated contraction of fetal middle cerebral arteries (Su et al., 2020).

Other animals can also be used in prenatal hypoxia studies. For chicks, prenatal hypoxia can also lead to memory deficits (Camm et al., 2001, 2005). For rabbits, uterine ischemia lasting 30, 37, 38, or 40 min at 21–22 days gestation can lead to hypertonia and abnormalities in motor control (Derrick et al., 2004). For guinea pigs, prenatal hypoxia resulted in decreased survival of neurons in the cerebral cortex and decreased the density of NeuN-immunoreactive neurons in the CA1 region of the hippocampus (Blutstein et al., 2013; Chung et al., 2015).

## Consequences of Prnatal Hypoxia in Neural Systems

### Abnormal Brain Development

The brain has the highest aerobic metabolism of all body organs, resulting in the greatest demand for oxygen and nutrients (Koundal et al., 2014). The adult central nervous system (CNS) is sensitive to hypoxia due to the lack of sufficient antioxidant enzymes and substrate reserves required for anaerobic metabolism (Esih et al., 2017; Piesova and Mach, 2020). Although it is generally believed that cerebral ischemia and hypoxia have similar properties, the characteristic of ischemia is usually accompanied by reducing or blocking the blood flow in some brain regions and leading to irreversible neuronal damage, whereas hypoxia leads to increased cerebral blood flow, which may lead to permanent or reversible changes in neuronal function, depending on its severity (Miyamoto and Auer, 2000). The effects of hypoxia and ischemia on developing brain cells are diverse: from the loss of neurons and oligodendrocytes to gliosis, changes in cell differentiation, decreases in synapse formation, and changes in neurotransmitter levels, even leading to irreversible cell injury and cell death (Robinson et al., 2005; Zhang et al., 2013). Studies in rodents have shown that chronic hypoxia can lead to anatomical changes similar to those seen in preterm infants, such as volume loss, reduced myelination and ventricular enlargement (Mayoral et al., 2009). In fact, in embryonic guinea pigs with maternal hypoxia, neural density, as measured by NeuN immunoreactivity in the CA1 field, was significantly decreased and GFAP and NeuN were significantly reduced in the CA1 region in adulthood (Blutstein et al., 2013), which also indicated that prenatal hypoxia could exert effects on neuronal development in the long term.

### Impaired Synaptic Plasticity

In terms of cellular mechanisms, hippocampal synaptic plasticity, including long-term potentiation (LTP) and long-term depression (LTD), is thought to underlie certain types of learning and memory (Collingridge et al., 2004). Impairment of brain function due to prenatal hypoxia is associated with impaired neurotransmitter circuits and synaptic plasticity (Herlenius and Lagercrantz, 2004). In rat offspring subjected to maternal hypoxia at E14 (Embryonic day 14), a decrease in the total number of pyramidal cortical neurons and the density of fragile synaptopodin-positive dendritic spines were observed, which subsequently differentiated into V-VI layer and formed cortical microcolumn and formation of cortical columns affects the formation of cortical cell structure, neuronal plasticity and postnatal behavior, which demonstrates cortical dysfunction (Vasilev et al., 2016). In normal aging rats, the number of synaptopodin-positive dendritic spines decreased, which may be one of the reasons for the decline in cognitive ability in Alzheimer’s disease (Arnold et al., 2013). Moreover, prenatal hypoxia significantly interfered with the basic synaptic transmission of CA3-CA1 synapses, with a twofold decrease in hippocampal LTP, decline in the protein level of GluN2B and reduced positive dendritic spines in the CA1 region of the hippocampus (Zhuravin et al., 2019). The decrease in the number of dendritic spines in the hippocampal CA1 region may be related to changes in the entorhinal cortex, which is thought to be the earliest event in the development of Alzheimer’s disease in humans (Killiany et al., 2002; Nalivaeva et al., 2018).

### Dysfunction of Key Pathways in Brain

#### Hypoxia-Inducible Factor 1 Pathway

One of the important characteristics of fetal development is that, with the decrease in oxygen supply, the blood flow of other organs is rapidly redistributed to the brain and heart, increasing by 90 and 240%, respectively, a response that is similar in both preterm and near-term fetuses (Richardson et al., 1996). In general, the initial response to hypoxia at the cellular level leads to changes in the expression of hypoxia inducible factor (HIF), which regulates many genes, including almost all genes involved in the glycolysis pathway (Semenza, 1998). It helps organisms to improve their survival ability under hypoxia by promoting erythropoiesis, vasodilation and angiogenesis. Prenatal hypoxia has been shown to change the expression of HIF-1α in the brains of embryonic rats, depending on the developmental stage of the embryo (Royer et al., 2000). Short and prolonged hypoxia induces a biphasic response to CRHR1 mRNA characterized by an initial decrease followed by a sustained increase and activation of the HPA axis, inducing hypoxic disease and behavioral changes. In this process, HIF-1α plays an active control role at the CRHR1 promoter -p1218 to -p1140 (Shi et al., 2017). At E14, it was found that hypoxia during pregnancy increased HIF-1α; however, hypoxia on E19 did not lead to any response in the HIF-1α genes, which indicates the difference in adaptability to hypoxia in these developmental stages (Royer et al., 2000).

#### Inflammation/Immunological Pathway

Inflammatory mediators are multifunctional cytokines that may be synthesized and secreted by several CNS cell types, including microglia, astrocytes and neurons, of which interleukin (IL)-1β, tumor necrosis factor-α (TNF-α) and IL-6 are among the most typical early responses, playing both a normal CNS development and a brain response to various forms of injury important role (Saliba and Henrot, 2001). A population-based survey found that the level of IL-6 in cerebrospinal fluid after perinatal asphyxia correlated with the severity of neonatal hypoxic-ischemic encephalopathy (HIE), brain injury and neurological outcome, suggesting a role for IL-6 in neonatal hypoxic-ischemic brain injury (Martin-Ancel et al., 1997). Another investigation showed that preterm infants with MRI-defined brain white matter injury had higher levels of IL-6, IL-10, and TNF-α in the CSF than infants without such injury. Elevated cytokine levels in the CSF of infants with cerebral white matter injury suggest altered inflammatory homeostasis (Ellison et al., 2005). In addition, pregnant Sprague-Dawley (SD) rats were dissected, inhaled oxygen concentrations were reduced to 10% for 3, 5, or 7 min to induce hypoxia, and mild hypoxic ischemia (HI) resulted in elevated IL-1β and IL-6 but not TNF-α mRNA and protein in brain and blood samples of the offspring at postnatal day 30 (Driscoll et al., 2018). NF-κB is rapidly activated in neurons and glial cells after asphyxial injury, and NF-κB p65 was shown to be upregulated in the rat brain 10 min after perinatal asphyxia, leading to the induction of genes associated with the innate immune response (Lubec et al., 2002; Morales et al., 2011).

#### Neurotrophic Pathway

Neurotrophic signaling is critical to prenatal and postnatal brain development because it affects the neuronal development process and its response to perinatal stress (Giannopoulou et al., 2018). Members of the neurotrophin family, including brain-derived neurotrophic factor (BDNF), nerve growth factor (NGF), neurotrophin-3 (NT-3), and neurotrophin-4 (NT-4), play a basic role in brain function and neuroprotection (Hennigan et al., 2007). Downregulation of BDNF was detected in umbilical cord samples from perinatal asphyxia (PA) patients who developed schizophrenia in adulthood (Cannon et al., 2008). Unilateral uterine artery ligation of Dunkin-Hartley guinea pigs at 30–32 days of gestation revealed a decrease in the number of neuronal cells in the cerebral cortex associated with a decrease in BDNF protein (Chung et al., 2015). Intrauterine growth restricted (IUGR) cases are characterized by intrauterine hypoxia, and NGF decreased significantly in the IUGR group compared with the fetus at the appropriate gestational age (Malamitsi-Puchner et al., 2007). Another study suggested that NT3 and NT4 decreased after hypoxia ischemia, while NGF and BDNF increased (Nikolaou et al., 2006).

#### Amyloid-β Peptide Related Pathway

The current majority view is that cases of Alzheimer’s disease (AD) are caused by an interaction between genetic and environmental factors. Hypoxia is considered to be an important environmental factor influencing the development of AD. Exposure of pregnant APP(Swe)/PS1(A246E) transgenic mice to hypoxia during gestation days 7–20 revealed higher levels of amyloid precursor protein (APP), lower levels of the Aβ degrading enzyme neprilysin, increased Aβ accumulation in the brains of prenatal hypoxic mice, and neuropathological changes mainly showing increased tau phosphorylation and enhanced activation of astrocytes and microglia (Zhang et al., 2013). Jackson Black C57 mice were exposed to 3% CO_2_/9% O_2_ for 2 h on the 17th day of pregnancy, and the content of APP in the hippocampus decreased significantly (Golan et al., 2009). In addition, prenatal hypoxia (7% O_2_, 3 h, E14) in aged male Wistar rats (over 12 months) or adult male rats (3–4 months) resulted in reduced expression of cortical metallopeptidases endothelin-converting enzyme (ECE-1) and neprilysin (NEP), two enzymes that regulate certain neuropeptides and are major β-amyloid degrading enzymes (Dubrovskaia et al., 2009).

### Prenatal Hypoxia Affects Brain Function in Long Term

Hypoxia is one of the main causes of brain damage with long-lasting behavioral implications (Golan and Huleihel, 2006). Different brain development and behavioral defects may be directly related to different ages and injury sites after hypoxia injury during pregnancy, which is worthy of attention. The effects of prenatal hypoxia on offspring of different ages are summarized in Table 2.

**TABLE 2:** Prenatal hypoxia affects brain function in long term.

**Age test**	**Prenatal hypoxia conditions**	**Brain development**	**Behavior**	**References**
2 years	Not mentioned	Structural damage in the cortex and the white matter	Not mentioned	Martinez-Biarge et al., 2010
2 years	Not mentioned	Severe cognitive impairment, moderate to severe cerebral palsy and death	Not mentioned	Twomey et al., 2010
Between 2 and 18 years	Not mentioned	Not mentioned	ADHD	Zhu et al., 2016; Banhegyi et al., 2020
19-year follow-up of average age 23 years	Not mentioned	Not mentioned	Schizophrenia and other non-emotional psychoses	Zornberg et al., 2000
P2	Rats around E18 *via* endotracheal intubation, an aneurysm clip placed on the arteries, removed after 45 min	Lacking oligodendrocyte and cortical neurons, cell proliferation decreased, cell death increased, and the level of pro-inflammatory cytokine levels increased at P2	Impaired motor skills as young adults	Robinson et al., 2005
P2, P8, P15	The rat uterine horn containing the fetus was immersed in a 37°C water bath for 20 min at P0	Excessive apoptotic cell death in the first week of life, resulting in the loss of a small number of neurons in the neostriatum	Not mentioned	Van de Berg et al., 2002
First month of postnatal	Acute normobaric hypoxia on E14 or E18 (3 h at 7% O_2_) in rats	Hypoxia on days 14 showed lower body weights, later separation of the outer ear from the skin of the head, and later opening of the eyes, ear from the skin of the head and later eye opening, while hypoxia on day 18 showed no statistically significant delay.	Decreased learning ability and impaired long-term and short-term memory	Dubrovskaya and Zhuravin, 2010
6 weeks after birth	From day 4 to day 21 of gestation, rats were treated with hypoxia (10.5% O_2_)	Decreased brain weight	Spatial learning and memory deficits	Wei et al., 2016
130 days	Day 15–21 of pregnancy, rats were treated with 10.5% O_2_	Not mentioned	Delayed onset latency of male sexual behavior and decreased ejaculation frequency	Hermans et al., 1993
4–6 months	Mice were placed in a plexiglass chamber (filled with a mixture of 3% CO_2_ and 9% O_2_, balanced N_2_ gas) for 2 h	Modified offspring brain morphology	Motor disabilities and memory damage	Golan et al., 2004

For humans and newborns with HIE, MRI studies show structural damage in the cortex and white matter at the age of 2 years (Martinez-Biarge et al., 2010). In another study, perinatal hypoxic-ischemic injury resulted in severe cognitive impairment, moderate to severe cerebral palsy and death at the age of 2 years (Twomey et al., 2010). Studies have shown that hypoxia during pregnancy is closely related to children’s attention deficit hyperactivity disorder (ADHD) (Zhu et al., 2016; Banhegyi et al., 2020). After a 19-year follow-up of 693 men and women (average age 23) born between 1959 and 1966 who were evaluated in early childhood, researchers found that schizophrenia and other non-emotional psychoses were strongly associated with prior hypoxic-ischemic events (Zornberg et al., 2000).

For animals, a midline laparotomy was performed with pregnant Sprague-Dawley rats by endotracheal intubation around pregnancy day 18. Prenatal hypoxia leads to the loss of oligodendrocyte lineage cells and cortical neurons, increased cell death, reduced cell proliferation, elevated pro-inflammatory cytokine levels at P2, and impaired motor skills at a young age (Robinson et al., 2005). The fetus containing the uterine horn was immersed in a 37°C water bath for 20 min at P0, the number of TUNEL-positive cells in asphyctic rats was 40 and 45% on P8 and P15, respectively, and the total number of striatal neurons in asphyctic young rats decreased by 16% on the 21st day. These data suggest that asphyxia leads to excessive apoptotic cell death in the first week of life, resulting in the loss of a small number of neurons in the neostriatum (Van de Berg et al., 2002). With acute normobaric hypoxia on E14 or E18 (3 h at 7% O_2_), prenatal hypoxia can delay the establishment of physiological development and motor behavior in the first month after birth (Dubrovskaya and Zhuravin, 2010). Rats treated with hypoxia (10.5% oxygen) from day 4 to day 21 of pregnancy significantly decreased brain weight and spatial learning and memory deficits in offspring 6 weeks after birth (Wei et al., 2016). Pregnant Sprague-Dawley females were exposed to continuous hypoxia (10.5% O_2_ from 15 to day 21 of pregnancy), and prenatal hypoxia significantly delayed the onset latency of male sexual behavior and decreased ejaculation frequency in adult offspring (130 days) but did not show a significant increase in female sexual potential (Hermans et al., 1993). In addition, prenatal hypoxia can also lead to adult offspring (4–6 months) motor disabilities and memory damage (Golan et al., 2004).

### Abnormal Neurological Behaviors

#### Movement

Motor skills and coordination develop in the first month of life, such as the ability of the newborn to grasp a rotating bar or grid (Golan and Huleihel, 2006). Brain damage caused by hypoxia has a long-term effect on the behavior of offspring. Children who survived hypoxia-ischemia at delivery suffer from ADHD, executive functioning disorder and object recognition disorder (Delcour et al., 2012a; Smith et al., 2016; Piesova and Mach, 2020). In animal models, the effects of hypoxia on behavior are similar to those of ADHD. Animals often exhibit motor and learning disabilities, hyperactivity disorder, and decreased memory, socialization, and attention (Piesova and Mach, 2020). Studies have shown that after prenatal hypoxic-ischemic injury, rats exhibit defects in motor skills, including ataxia and delayed motor planning, similar to those of affected infants (Robinson et al., 2005). Although congenital motor response defects in neonatal rats after prenatal hypoxia became less obvious in the first month of life with the development of young rats, more skilled movements, such as reaching and pushing, and learning new motor reflexes, remained compromised in adulthood (Zhuravin et al., 2002).

#### Memory

Different from those conditions where pups actually compensated and restored during the first month of development, cognitive impairment caused by prenatal hypoxia at E14 or E18 can still be detected at all testing stages after birth (Nalivaeva et al., 2018). The hippocampus is a part of the limbic system that originates in the medial telencephalon and plays an important role in the encoding of information; it is related to long-term and short-term memory and spatial navigation (Khalaf-Nazzal and Francis, 2013). Prenatal hypoxia causes impairment of different types of memory (short-term memory and long-term memory, working memory) in rat offspring, which is associated with the structural changes observed in the hippocampus (Zhuravin et al., 2011; Nalivaeva et al., 2012; Cunha-Rodrigues et al., 2018). Exposure of pregnant rats to 10.5% oxygen from day 15 to day 21 of gestation resulted in increased matrix metalloproteinase activity and significant cell death in the hippocampal region of neonatal rats, which was associated with worse neurobehavioral outcomes (Tong et al., 2010). On the other hand, learning deficits in the offspring of adult pregnant rats exposed to hypobaric hypoxia during gestational days 10–20 correlated with a significant reduction in the frequency of polyplasmic neurons in the subdentate region (Foley et al., 2005).

#### Emotion

Prenatal hypoxia not only changes the behavior of movement and memory but also affects emotion. Pregnant rats during gestational days 19–20 were exposed to low oxygen content (10.5% O_2_) for 4 h per day, and prenatal hypoxic rats had a higher response to the stress stimulus, leading to anxiety- and depression-like behaviors in the offspring (Sedlackova et al., 2014). Animals subjected to asphyxia showed a significant decrease in social aggression, an increase in social contact behavior, and an increase in anxiety levels (Weitzdoerfer et al., 2004). Prenatal hypoxia also resulted in decreased social interaction, impaired male sexual behavior, and decreased acquisition of passive and active avoidance responses (Dubrovskaya and Zhuravin, 2010). In addition, prenatal hypoxia is closely related to schizophrenia (Howell and Pillai, 2016). Interestingly, some early studies have shown that hypoxia during pregnancy does not affect anxiety-like behavior in rats with prenatal hypoxia (Buwalda et al., 1995), while rats exposed to prolonged hypoxia (15 and 20 min of asphyxia) showed a decrease in anxiety-related behavior in the elevated plus maze (Hoeger et al., 2000). These opposite results may be caused by the use of different models and different temporal checkpoints in these studies.

## Possible Mechanisms Underlying the Neurological Disorders Caused by Prenatal Hypoxia

Although the phenomenon of nervous system development disorders and psychiatric disorders due to prenatal hypoxia has been continuously confirmed, there is no complete and systematic theoretical system of its occurrence. At present, there are many hypotheses about the mechanism: abnormal epigenetic modification, endocrine axis dysfunction, oxidative damage and mitochondrial dysfunction.

### Abnormal Epigenetic Modification

Epigenetic modification is a form of gene regulation of a specific genome that does not change the sequence of DNA nucleotides, but can be stably inherited through a variety of mitotic modes (Bernstein et al., 2007). Epigenetic modifications include DNA methylation, histone modification and non-coding RNA regulation. Epigenetic modification allows phenotypic plasticity, allowing individuals to regulate gene expression through environmental changes, which is a heritable change (Boland et al., 2014). This pattern of gene regulation has attracted increasing attention in development programs. The early adverse intrauterine environment may produce abnormal epigenetic markers, which may lead to metabolic syndrome and other chronic adult diseases after birth (O’Sullivan et al., 2012). Oxygen levels through HIF1α-Notch signaling interactions and DNA demethylation of astrocytic genes epigenetically regulate the fate of neural precursor cells (Mutoh et al., 2012). In the normally developing brain, hypoxic culture conditions (<5%) can promote the differentiation of neural precursor cells into astrocytes at midgestation by activating the Notch signaling pathway and upregulating the transcription factor nuclear factor IA (NFIA), which leads to the demethylation of astrocyte-specific genes such as S100β and GFAP; however, under normoxic conditions, these processes are inhibited (Mutoh et al., 2012). It is reasonable to suggest that a lower oxygen level will promote the differentiation of astrocytes in the developing brain even more strongly, resulting in a decrease in the ratio of neurons to astrocytes. Maternal hypoxia (10.5% O_2_, E15-21) leads to a decrease in glucocorticoid receptor expression in the developing rat brain, and decreased GR expression is attributed to increased DNA methylation, reduced binding of transcription factors Sp1 and Egr-1 to GR gene exon 1_11_ and 1_7_ promoters, and decreased GR exon 1_11_ and 1_7_ mRNA variants (Gonzalez-Rodriguez et al., 2014). Gestational intermittent hypoxia (4 h per day, E1–E21, 10.8% O_2_) induced demethylation of the CPG site of Crhr1 DNA in the promoter in male offspring of Sprague-Dawley rats, which triggered an increase in Crhr1 mRNA expression and anxiety-like behavior in adult offspring (Wang et al., 2013). Three-month-old APP(swe)/PS1(dE9) mice exposed to hypoxia for 6 h/day for 30 days exhibited aggravated AD progression, accompanied by decreased genomic DNA demethylation and DNA methyltransferase 3B (DNMT3b) expression. DNMT inhibition increased amyloid precursor protein and β- and γ-secretase protein expression. In contrast, overexpression of DNMT3b inhibited their levels *in vitro* (Liu et al., 2016).

Studies have confirmed the role of a unique class of small non-coding microRNAs (miRNAs) in the post-transcriptional regulation of target genes in response to hypoxia (Nguyen et al., 2013). The mechanism of miRNA production and maturation depends on the oxygen supply to the cells, and the regulation of HIF expression depends on the type of miRNA (Taguchi et al., 2008; Liu et al., 2015). Since REST inhibits the expression of neuron-specific proteins and miRNA genes, under hypoxic conditions, the REST content decreases; therefore, reducing the oxygen supply plays a regulatory role in maintaining the miRNA profile of neurons (Liang et al., 2014). In the process of hypoxia-induced embryonic brain cortical apoptosis and neuronal apoptosis, the transcription factor c-myc plays an inhibitory role in regulating the expression of the miR-23b-27b cluster during hypoxia (Chen et al., 2015). In addition, oxygen tension is associated with histone H3 methylation and coactivator recruitment, chromatin transcriptionally active (euchromatin) or inactive (heterochromatin) depends on histone modifications, and prenatal hypoxia can directly or indirectly inhibit the expression of G6Pase through the increase of histone H3 [K9] methylation (Osumek et al., 2014).

### Endocrine Axis Dysfunction

Intrauterine programming refers to the critical stage of development under unsatisfactory intrauterine conditions (such as hypoxia), which will have irreversible consequences (Sedaghat et al., 2015). The developmental programming hypothesis of the intrauterine endocrine axis is one of the most accepted hypotheses. The endocrine axis mainly includes the hypothalamic-pituitary-adrenal (HPA) axis, growth hormone (GH)-insulin-like growth factors (IGFs) and the renin-angiotensin system (RAS) (Fowden and Forhead, 2004; Fowden et al., 2005). The HPA axis is one of the most important neuroendocrine axes and plays an important role in the stress defense response before and after birth. Fetal overexposure to maternal glucocorticoids may be a major initial factor in altering fetal HPA axis programming, and these changes will increase the susceptibility of adult neuropsychiatric disorders, such as schizophrenia and depression, as well as metabolic diseases (Zhang et al., 2014).

It has been shown that glucocorticoids play an important role in neurological function and that high concentrations of glucocorticoids can exacerbate excitotoxic effects and promote neuronal death (Stein-Behrens et al., 1992; Lee et al., 2002; Dumas et al., 2010). Severe prenatal hypobaric hypoxia leads to a persistent increase in the baseline blood levels of corticosterone in adult and aged rats, and the total amount and nuclear localization of GR in the hippocampus of newborn rat pups decrease significantly and persist throughout life (Vetrovoy et al., 2020). Clinical studies have shown that the volume of cortical gray matter in preterm infants treated with synthetic glucocorticoids is reduced and that their long-term neuromotor and cognitive functions are impaired (Murphy et al., 2001; Yeh et al., 2004).

### Oxidative Damage and Mitochondrial Dysfunction

Oxygen is the terminal receptor of electrons in the mitochondrial electron transport chain and is associated with oxidative phosphorylation and energy production through ATP synthesis. If the oxygen supply is reduced or completely blocked, electron transport is slowed down, which will not fully meet the metabolic needs of the cell (Piesova and Mach, 2020). Hypoxia also increases the level of reactive oxygen species (ROS) because it disrupts complexes I and III of the mitochondrial electron transport system, and in a hypoxic environment, the electron flow is slow, so the possibility of superoxide anion formation increases (Poyton et al., 2009). ROS destroy membrane components, induce neuronal apoptosis and damage the cerebrovascular system (Koundal et al., 2014). Prenatal hypoxia also leads to increased placental oxidative stress and decreased mitochondrial respiration and mitochondrial fusion, which may lead to disorders of placental function and fetal development (Ganguly et al., 2021). Increased oxidative stress also leads to damage to proteins, lipids and DNA, which disrupts normal cellular function (Silvestro et al., 2020). Rapid re-oxygenation after hypoxia can disrupt the fetal blood-brain barrier by producing ROS, thereby amplifying the effects of molecules in fetal blood on the brain (Piesova and Mach, 2020).

## Future Prospects

Although the molecular mechanisms of brain development and functional damage caused by prenatal hypoxia have been deeply studied, all the changes in molecules and behavior caused by prenatal hypoxia are not yet fully understood. Further studies of prenatal hypoxia models will enable us to better understand the mechanism of neuron dysfunction in fetal development and design new prevention strategies to restore brain integrity and normal behavior. Clinically, the use of ultrasound, Doppler velocimetry and antenatal screening for high-risk pregnancies can help identify at-risk fetuses. Interventions such as intrauterine resuscitation or surgical delivery may reduce the risk of severe hypoxia due to intrauterine injury and improve the long-term neurological prognosis.

A number of pharmacological approaches can also be used in pregnant women during pregnancy, and magnesium sulfate (MgSO_4_) is administered to pregnant women with premature birth to prevent the development of cerebral palsy in the infant (Shepherd et al., 2017). Animal studies further showed that MgSO_4_ was administered to pregnant mice on day 17 of gestation, 4 h before chamber hypoxia (9% O_2_, 2 h), reduced motor deficits in the offspring and protected their developing Purkinje cells in the postnatal period (Golan et al., 2004). In a prenatal hypoxic guinea pig model, treatment of hypoxic pregnant female with MgSO_4_ attenuated the hypoxia-induced increase in Ca(2+) inward flow in neuronal nuclei of the guinea pig fetus and protected the nuclear membrane function of neuronal cells (Maulik et al., 2005). Maternal MgSO_4_ treatment also reversed the hypoxia-induced GABA deficit pathway in neonates and reversed the hypoxia-induced loss of inhibitory neuronal subpopulations in neonates (Louzoun-Kaplan et al., 2008). Pretreatment with MgSO_4_ reduces hypoxia-induced motor deficits in a mouse model of maternal hypoxia and prevents the short-term memory impairment but not long-term memory impairment (Golan et al., 2004). In a rat model of prenatal hypoxia, nimodipine was shown to prevent hypoxia-induced brain growth inhibition in offspring (Nyakas et al., 1994). In addition, valproic acid restored neprilysin (NEP) activity and memory deficits in adult offspring rats caused by prenatal hypoxia, which involves regulating the NEP gene by binding the APP intracellular domain (AICD) to the NEP promoter (Nalivaeva et al., 2012). For memory impairment in hypoxic offspring during pregnancy, it has been shown that maternal vitamin C supplementation during hypoxic gestation can prevent excessive oxidative stress in the placenta, thereby ameliorating the adverse effects of prenatal hypoxia on hippocampal tissue atrophy and memory loss in adult offspring (Camm et al., 2021). Crocin significantly improves hypoxia in the brain tissue of neonatal rats during pregnancy by improving memory impairment and molecular alterations related to hypoxia (Ghotbeddin et al., 2021).

Some gene therapy methods are also used for hypoxia during pregnancy. Prenatal hypoxia (7% O_2_, 3 h) in rats on the 14th day of embryonic development increases caspase-3 content and activity while decreasing the levels of NEP and AICD, which regulates NEP expression. Increased levels of AICD and NEP were found in hypoxic offspring after a single injection of a caspase inhibitor (i.v., Ac-DEVD-CHO0) (Kozlova et al., 2015). Defective trophoblastic invasion observed in preeclampsia (PE) or fetal growth restriction (FGR) is thought to result in relative placental hypoxia. Tadalafil, a selective phosphodiesterase 5 inhibitor, reduced the expression of HIF-2α in the placenta and in the brain of FGR fetuses, and *in utero* treatment showed improved synaptogenesis and myelination in FGR offspring at P15 and P30 (Tachibana et al., 2019). Selective head cooling (34.5°C) and whole-body cooling (33.5°C) are two hypothermic treatments commonly used as protective therapy for moderate to severe HIE, within the first 6 h after birth and maintained for 3 days to effectively reduce brain damage (Silveira and Procianoy, 2015). Maternal voluntary exercise during pregnancy can increase the number of hippocampal neurons and angiogenesis and protect against mild chronic postnatal hypoxia in rat offspring (Akhavan et al., 2012). In addition, stem cells, erythropoietin, allopurinol, flunarizine, nitric oxide, and other traditional Chinese medicines and related extracts are also involved in the treatment of HIE (Zhao et al., 2016).

## Author Contributions

MS conceived and supervised the project. BW, HZ, and JL wrote the manuscript. All authors contributed to the article and approved the submitted version.

## Conflict of Interest

The authors declare that the research was conducted in the absence of any commercial or financial relationships that could be construed as a potential conflict of interest.

## Publisher’s Note

All claims expressed in this article are solely those of the authors and do not necessarily represent those of their affiliated organizations, or those of the publisher, the editors and the reviewers. Any product that may be evaluated in this article, or claim that may be made by its manufacturer, is not guaranteed or endorsed by the publisher.
